# Rheological Abnormalities in Human Erythrocytes Subjected to Oxidative Inflammation

**DOI:** 10.3389/fphys.2022.837926

**Published:** 2022-02-23

**Authors:** Toru Maruyama, Michinari Hieda, Shiro Mawatari, Takehiko Fujino

**Affiliations:** ^1^Department of Hematology, Oncology and Cardiovascular Medicine, Kyushu University Hospital, Fukuoka, Japan; ^2^Institute of Rheological Function of Foods Co., Ltd., Hisayama, Japan

**Keywords:** erythrocytes, rheology, oxidative inflammation, membrane, eryptosis

## Abstract

Erythrocytes are oxygen carriers and exposed to redox cycle in oxygenation and deoxygenation of hemoglobin. This indicates that circulating erythrocytes are vulnerable to the oxidative injury occurring under the imbalance of redox homeostasis. In this review article, two topics are presented concerning the human erythrocytes exposed to the oxidative inflammation including septic and sterile conditions. First, we demonstrate rheological derangement of erythrocytes subjected to acute oxidative injury caused by exogenous generators of reactive oxygen species (ROS). Erythrocyte filterability as whole-cell deformability has been estimated by the gravity-based nickel mesh filtration technique in our laboratory and was dramatically impaired in a time-dependent manner after starting exposure to the ROS generators, that is associated with concurrent progression of membrane protein degradation, phospholipid peroxidation, erythrocyte swelling, methemoglobin formation, and oxidative hemolysis. Second, we introduce an impairment of erythrocyte filterability confirmed quantitatively in diabetes mellitus and hypertension of animal models and patients under treatment. Among the cell geometry, internal viscosity, and membrane property as the three major determinants of erythrocyte deformability, erythrocyte membrane alteration is supposed to be the primary cause of this impairment in these lifestyle-related diseases associated with persistent oxidative inflammation. Excessive ROS trigger the inflammatory responses and reduce the erythrocyte membrane fluidity. Oxidative inflammation increasing erythrocyte membrane rigidity underlies the impaired systemic microcirculation, which is observed in diabetic and/or hypertensive patients. On the other hand, elevated internal viscosity caused by sickle hemoglobin polymerization is a primary cause of impaired erythrocyte filterability in sickle cell disease (SCD). However, oxidative inflammation is also involved in the pathophysiology of SCD. The physiologic level of ROS acts as signaling molecules for adaptation to oxidative environment, but the pathological level of ROS induces suicidal erythrocyte death (eryptosis). These findings provide further insight into the ROS-related pathophysiology of many clinical conditions.

## Introduction

Reactive oxygen species (ROS) such as superoxide anion (O_2_^•⁣–^) and hydroxyl radical (^•^OH) are inevitable byproducts of respiratory and metabolic pathways in living cells including circulating erythrocytes that bind, carry, and deliver molecular oxygen (O_2_). In contrast, intrinsic antioxidative enzymatic (superoxide dismutase, catalase, glutathione peroxidase, etc.) and non-enzymatic (α-tocopherol, etc.) defense mechanisms scavenge ROS generated in erythrocytes to survive oxidative stress. However, ROS accumulate under pathological conditions out of cellular redox control. Erythrocytes are a primary target of oxidative stress because hemoglobin is an iron-containing protein. Under the cyclic oxygenation and deoxygenation of hemoglobin, heme iron mediates hydroxyl radical generation *via* iron-catalyzed Fenton reaction as follows (Sadrzadeh et al., 1984):


(1) O+2•-Fe→3+Fe+2+O2

(2) 2O+2•-2H→+HO2+2O2

(3) HO2+2Fe→2+OH•+OH+-Fe+3

Iron generates ROS by self-cycling between ferrous (Fe^2+^) and ferric (Fe^3+^) states. According to the ROS-induced denaturation of hemoglobin, heme further releases free iron that exerts catalytic actions on various cellular components, and ROS are also generated by degradation of heme (Tsamesidis et al., 2020). Once the release of reactive iron reaches the threshold level, self-regenerating auto-catalytic reaction proceeds and causes erythrocyte membrane perturbation including membrane protein degradation and phospholipid peroxidation.

Inflammation provides a defensive mechanism to foreign pathogens or intrinsic ROS-injured cellular components. Growing evidence suggests that ROS act as signaling molecules to restore the damaged cellular components and eliminate necrotic or apoptotic cells leading to an inflammatory response. As an example, systemic inflammation is found in sepsis as a clinical syndrome secondary to severe infection. Sepsis is sometimes associated with shock that is characterized by hemodynamic instability, impaired systemic microcirculation causing hypoxia and acidemia, multiple organ failure leading to high morbidity. These clinical findings are based on abnormal hemorheology including rigid erythrocytes, activated platelets, adhesive leukocytes, excessive inflammatory cytokines, and oxidative stress (Bateman and Walley, 2005). There are multiple sources of ROS generation including activated neutrophils, endothelial cells, erythrocytes *per se*, plasma xanthine oxidase, and oxygen therapy to improve hypoxia (Bar-Or et al., 2015).

Persistent oxidative stress promotes sterile inflammation to restore the cell and tissue damage, and chronic inflammation plays an important role in the pathophysiology of many clinical conditions such as type 2 diabetes mellitus, hypertension (Mittal et al., 2014), and sickle cell disease (SCD), a hereditary hemoglobin disorder (Nader et al., 2020). Circulating erythrocytes are exposed to ROS and inflammatory cytokines under the chronic inflammation associated with unstable redox homeostasis. Inflammatory cytokines, such as transforming growth factor-β1 and endothelin-1, activate NADPH oxidase and increase superoxide (O_2_^•⁣–^) generation as follows (Dammanahalli and Sun, 2008; Peshavariya et al., 2014):


(4) NADPH+2O→2NADP++2O2•-+H+

Furthermore, upregulation of redox-sensitive transcriptional factors such as nuclear factor κ-B (NFκ-B) expresses some genes involved in inflammatory pathways and accelerates systemic inflammation (El Assar et al., 2013). These indicate that erythrocytes are vulnerable to persistent oxidative inflammation. This article reviews the rheological abnormalities in erythrocytes as a model of cellular response to acute oxidative inflammation and introduces the rheological behaviors of erythrocytes in ROS-associated clinical settings such as diabetes, hypertension, and SCD.

## Estimation of Erythrocyte Deformability

Circulating erythrocytes deform to transit through the microcirculation, because the diameter of normal erythrocytes (6–8 μm) is greater than the minimum diameter of capillary network (3–4 μm). Apparent blood viscosity decreases with decreasing microvascular diameter, which is known as the Fahraeus–Lindqvist effect (Fahraeus and Lindqvist, 1931). Because erythrocytes deform as bullet-like axisymmetric configurations, that is confirmed by a high-speed camera and image analysis (Jeong et al., 2006; Tomaiuolo et al., 2009). The erythrocyte deformation yields a cell-free layer at the microcapillary wall and a reduction in local hematocrit. The ability for erythrocytes to deform is termed deformability. Reduction of apparent blood viscosity is attributed to the concert of the cell-free layer width, local hematocrit reduction, and erythrocyte deformability. Ektacytometry, a standard technique to investigate this deformability, enforces the elongating deformation upon erythrocytes suspended in a high viscosity medium by applying the known shear stress. A group of passed and elongated erythrocytes creates a specific diffraction pattern captured by scattered light and a video camera. The elongation index is calculated by the short and long axes of the ellipsoidal laser beam diffraction pattern as an index of erythrocyte deformability (Piety et al., 2021). Elongation of erythrocytes under the fixed shear condition is quantified, and a steady-state shear-deformation relationship is estimated, which is a great merit of ektacytometry. However, the recent finding of the erythrocyte deformation is shear-dependent shape transitions, i.e., erythrocytes demonstrate first tumbling, then rolling, and finally, polylobed shape with an increase in shear rate under the physiological medium conditions (Lanotte et al., 2016; Mauer et al., 2018) and such dynamic morphological transition governs the shear thinning in physiological microcirculation. These findings limit the physiological relevance of elongating erythrocyte deformation observed in the high viscosity medium of ektacytometry.

**FIGURE 1:**
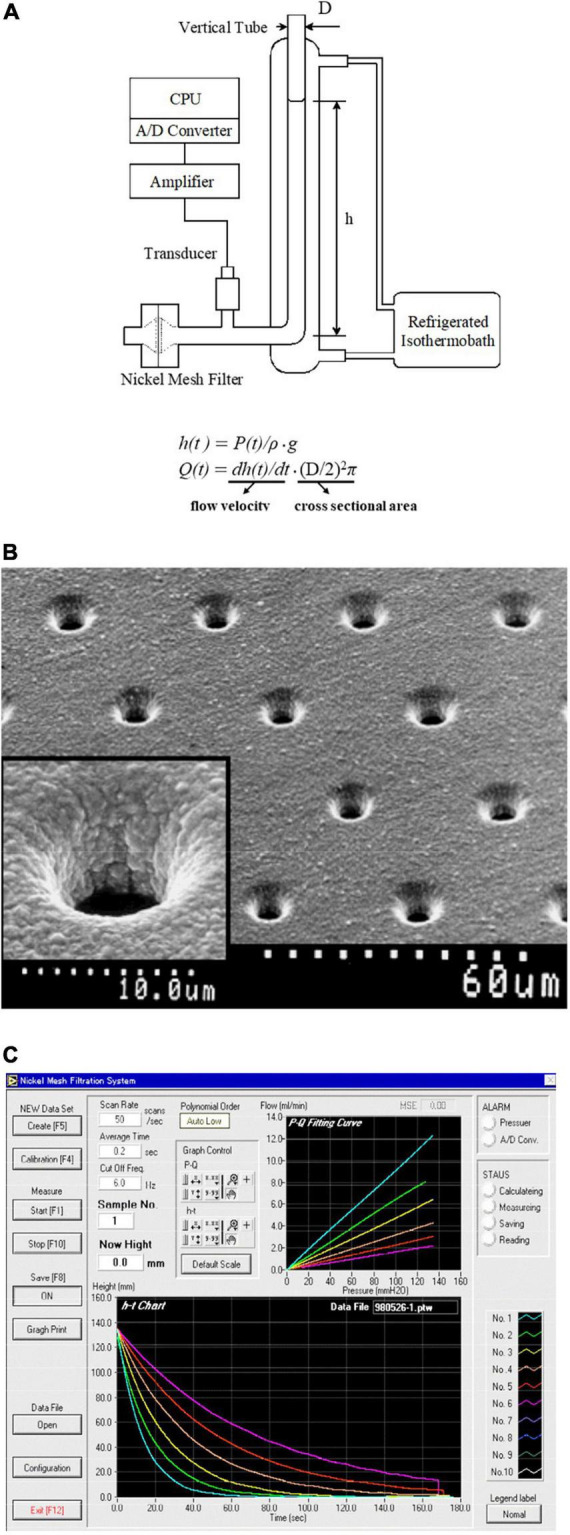
Principle and scheme of the nickel mesh filtration technique. **(A)** Schematic illustration of the gravity-based filtration apparatus using a nickel mesh filter. Hydrostatic pressure [*P(t)*; mmH_2_O] was monitored during continuous filtration by gravity using a pressure transducer. *P(t)* was converted to height [*h(t)*; mm] of the meniscus within the vertical tube using the equation of *h(t)* = *P(t)/*ρ*g*. The flow rates [*Q(t)*; ml/min] was obtained by multiplying the first-time derivative of *h(t)* [*dh(t)/dt*], rate of fall of the meniscus, by the internal cross-sectional area of the vertical tube, i.e., *Q(t)* = *dh(t)/dt* ⋅ (D/2)^2^π, where D, the internal diameter of the vertical tube; *g*, acceleration of gravity; ρ, the specific gravity of specimens. **(B)** Scanning electron microscopic photographs of a nickel mesh filter. The inset shows a magnification of a single pore. Calibrations are indicated at the bottom. **(C)** The main window of the computer screen shows the relationships of *P(t)-Q(t)* (upper) and *h(t)-t* (lower). The filtration material is dextran sulfate solution at various concentrations. Linear *P(t)-Q(t)* relationships indicate that dextran sulfate solution is a Newtonian fluid.

The deformability is a fundamental rheological function of the erythrocyte population. The lifespan of human healthy erythrocytes is about 120 days, and circulating erythrocytes show individual aging. Senescent erythrocytes are dense, shrunk, dehydrated, and less deformable, while the surface-area-to-volume ratio (sphericity) shows no significant changes during aging (Waugh et al., 1992). The least deformable erythrocytes are removed finally by the spleen-resident macrophages. Therefore, the deformability of the erythrocyte population is heterogeneous, and modern technologies and innovations have made it possible to investigate the distribution of age-dependent erythrocyte deformability (Dobbe et al., 2002; Depond et al., 2020) and shear modulus (Saadat et al., 2020). On the other hand, practical measurement of mean erythrocyte deformability in many clinical samples is also important. One of the recent techniques drawing attention is the microfluidic assessment of erythrocyte-mediated microcapillary occlusion assessed by monitoring electrical impedance, which is linked to the pathophysiology and therapeutic responsiveness of SCD (Man et al., 2021a,b). Gravity-based filtration technique using a thin nickel mesh filter has been used in our laboratory for deformability measurement under physiological conditions (Figure 1). Nickel mesh filter produced by photofabrication technique is characterized by highly uniform pore size, shape, and distribution. This filtration apparatus demonstrates the relationship between pressure (*P*) and flow rate (*Q*) of erythrocytes suspended in physiological saline with hematocrit adjusted exactly to 3.0% (Odashiro et al., 2015; Arita et al., 2020). Very low hematocrit allows independent behaviors of individual erythrocytes without aggregation. Deformability estimated by this filtration technique is the average of the entire erythrocytes in suspension. However, this method is practical, cost-effective, physiologically relevant, and based on the fundamental *P*-*Q* relationship fitted by the laminar fluid flow model (*P*-*Q* curve). The common features of the microfluidic erythrocyte occlusion and nickel mesh erythrocyte filtration are cost-effectiveness and functional assays of erythrocytes rheology without high-resolution imaging. Considering a small fraction (1–5%) of abnormal erythrocyte subpopulation affecting the whole blood fluid behavior (Kuck et al., 2022), the microfluidic or filtration process is influenced by the least deformable erythrocytes, whereas it is a small doubt whether such small subpopulation creates its specific diffraction pattern in ektacytometry (Piety et al., 2021).

## Effects of Oxidative Stress on Erythrocyte Deformability

Erythrocyte deformability plays a pivotal role in microcirculation, and the major determinants of the deformability are (1) cell geometry, (2) internal viscosity, and (3) membrane properties of circulating erythrocytes (Mohandas and Chasis, 1993). Biconcave disk configuration, low internal viscosity, and viscoelastic membrane property realize well deformable erythrocytes at a whole-cell level. Sepsis as a model of serious infection-induced inflammation impairs erythrocyte deformability. Rather, the impaired deformability is an early sign and prognostic marker of critically ill patients including those with sepsis (Totsimon et al., 2017). Impaired deformability is associated with multiple organ failure and life prognosis, and rigid erythrocytes are attributed to the marked oxidative stress generating ROS, reduced antioxidative capacity, and disrupted internal Ca^2+^ homeostasis (Bateman et al., 2017). Microvascular dysfunction in sepsis induces erythrocyte capillary flow stopping that reduces systemic functional capillary density, tissue hypoxia-reoxygenation insult, and subsequent inflammation. This phenomenon augments ROS generation leading to the further impairment of erythrocyte deformability (Bateman and Walley, 2005).

**FIGURE 2:**
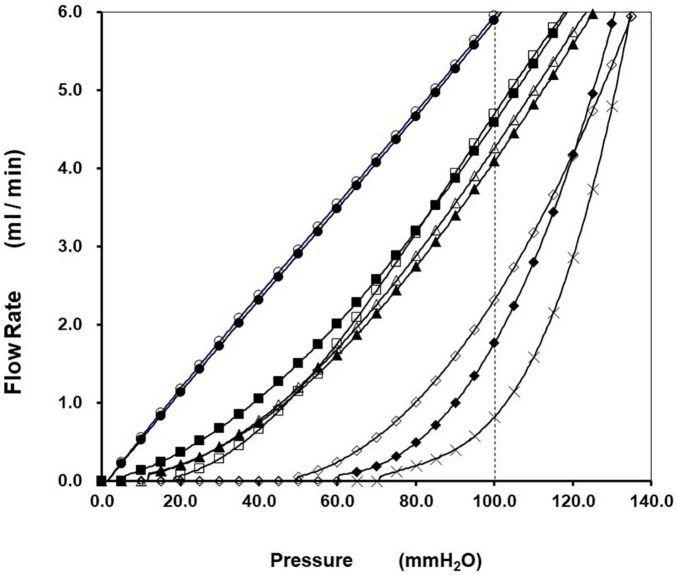
Impairment of erythrocyte filterability caused by 2,2′-azobis(2-amidinopropane) dihydrochloride (AAPH). Representative relationships of *P(t)-Q(t)* during continuous filtration using HEPES-buffered control saline and human erythrocyte suspensions obtained from healthy volunteers are presented. The hematocrit of erythrocyte suspension was 3.0%, and the pore size of the nickel mesh filter was uniformly 5.31 μm. The *P(t)-Q(t)* relationships correspond to the two passages of control saline (◯, 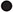) and sequential passages of erythrocyte suspensions in preincubation (□, ■) and under the incubation with 50 mM AAPH at incubation times of 20 (△), 40 (▲), 60 (◇), 120 (◆), and 180 (X) min at 36°C, respectively. Linear *P(t)-Q(t)* relationships passing origin indicate filtration of control saline behaving as a Newtonian fluid. The superimposable linearity satisfies the reproducibility of this filtration system. Continuous filtration of erythrocyte suspensions before incubation produced linearity at a high-pressure region that is followed smoothly by the concaved curve at a low-pressure region that is compatible with non-Newtonian behavior. The *P(t)-Q(t)* curves shifted to the rightward according to the progression of incubation. Filterability of erythrocyte suspensions (%) was defined as *Q(t)* of suspension at *P(t)* of 100 mmH_2_O (vertical dotted line) divided by *Q(t)* of saline at the same *P(t)*. Marked suppression of erythrocyte filterability yields a no-flow phenomenon under the pressure of 70 mmH_2_O (intercept pressure) 180 min after starting incubation [cited from Odashiro et al. (2014) with permission and minor modification].

**FIGURE 3:**
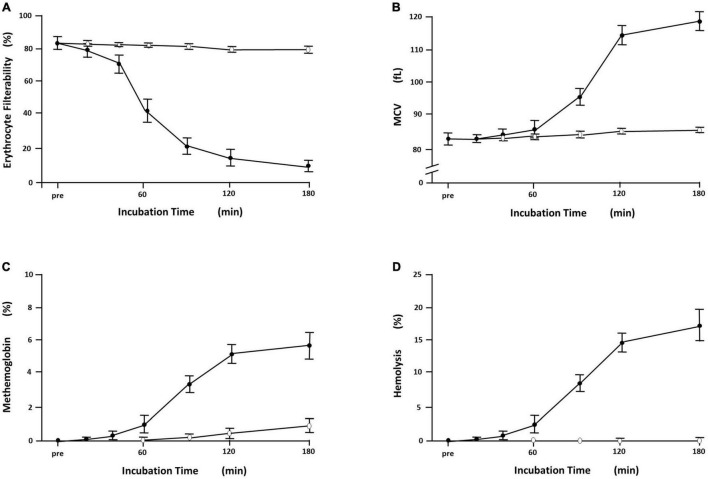
Time-dependent derangement of erythrocytes exposed to 2,2′-azobis(2-amidinopropane) dihydrochloride (AAPH). Symbols and bars indicate mean ± SD (*n* = 6). **(A)** The filterability of erythrocytes exposed to 50 mM AAPH at 36°C showed time-dependent reduction (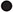), which was marked at incubation time segment of 40–60 min after starting incubation, whereas the filterability of erythrocyte suspension without exposure to AAPH remained at the preincubation levels around 80% (◯). **(B)** Time-dependent changes of the mean corpuscular volume (MCV; fl) of erythrocytes with and without exposure to 50 mM AAPH. MCV is calculated automatically by hemocytometer as a surrogate of cell size. Erythrocytes exposed to 50 mM AAPH showed a sigmoidal increase in MCV (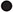), which was evident 60 min after starting incubation at 36°C. Erythrocytes without exposure to AAPH (◯) showed no discernible changes in MCV. **(C)** Time-dependent increases in methemoglobin formation of erythrocytes (%) with and without exposure to 50 mM AAPH. Methemoglobin was assayed by standard spectrophotometric methods using absorbance differences of hemolysate in the presence and the absence of potassium cyanide and/or potassium ferricyanide at the wavelength of 630 nm. Erythrocytes exposed to AAPH showed a time-dependent sigmoidal increase in methemoglobin formation (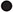), which was evident 60 min after starting incubation at 36°C. Erythrocytes without exposure to AAPH (◯) showed slight methemoglobin formation due to natural oxidation. **(D)** Hemolytic time course of erythrocyte suspension (%) exposed to 50 mM AAPH. Hemolysis was quantified by the absorbance of hemoglobin at 540 nm in the supernatant, and percent hemolysis (%) was calculated with comparison to complete hemolysis using distilled water. Erythrocytes exposed to AAPH (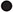) showed time-dependent hemolysis, which was evident 60 min after starting incubation at 36°C, whereas erythrocyte suspension without exposure to AAPH (◯) showed no evident hemolysis [cited from Odashiro et al. (2014) with permission].

Sterile inflammation after oxidative damage is a ubiquitous biological reaction, and a ROS generator is often applied to erythrocyte suspension as an *ex vivo* oxidative injury model. Our laboratory confirmed that erythrocyte deformability is suppressed dramatically by acute oxidative stress generated by 2,2′-azobis(2-amidinopropane) dihydrochloride (AAPH), *tertiary-*butyl hydroperoxide (tBHP), and superoxide anion (O_2_^•⁣–^) produced by hypoxanthine-xanthine oxidase reaction (Uyesaka et al., 1992; Okamoto et al., 2004; Odashiro et al., 2014). AAPH-induced time-dependent suppression of the deformability is demonstrated in Figure 2. *P*-*Q* curve shifting to the rightward indicates that erythrocyte filterability was impaired significantly according to the progression of incubation. No flow phenomenon was observed 180 min after starting incubation under the positive pressure of 70 mmH_2_O, indicating that rigid erythrocytes completely obstruct all the nickel mesh pores. AAPH impairs the filterability greatly in a time-dependent sigmoidal manner (Figure 3A). AAPH-induced impairment of deformability is suspected to be attributable to changes in any determinant (geometry, internal viscosity, and membrane property). For the cell geometry, the mean corpuscular volume of erythrocytes (MCV; fL) as a measure of cell size increases time-dependently after acute exposure to 50 mM AAPH. This swelling is accelerated 60 min after starting exposure (Figure 3B). ROS-induced erythrocyte swelling was confirmed also in the case of 0.4 mM tBHP application (Okamoto et al., 2004) and hypoxanthine-xanthine oxidase reaction liberating superoxide anion (Uyesaka et al., 1992) in the Ca^2+^-free condition.

Biological response to oxidative stress has been studied extensively using oxidative agents. The application of these agents to intact erythrocytes provides a cellular model of acute oxidative inflammation and premature erythrocyte senescence (Mohanty et al., 2014). Phenazine methosulfate [C_14_H_14_N_2_O_4_S; MW = 306.34] acts as an NADPH oxidant generating superoxide (O_2_^•⁣–^) intracellularly and causes internal oxidation (Prema and Gopinathan, 1974). Phenazine methosulfate-treated erythrocytes are shrunk in a Ca^2+^-containing medium, whereas they are swollen in a Ca^2+^-free medium (Lisovskaya et al., 2008). These findings are explained by the activation of Ca^2+^-activated K^+^ channels (Gardos channels) causing cell dehydration and shrinkage (Gardos, 1958). AAPH [C_8_H_18_N_6_⋅2HCl; MW = 271.19] and tBHP [(CH_3_)_3_COOH; MW = 90.12] are oxidative agents generating alkyl radicals in a time- and dose-dependent manner. AAPH is a representative hydrophilic azo compound, and tBHP is an amphiphilic organic compound. These agents liberate alkyl radicals by thermolysis or photolysis. Extracellular alkyl radical attacks erythrocyte surface membrane leading to the peroxyl radical generation and membrane phospholipid peroxidation. Phospholipid peroxyl radical propagates to the adjacent membrane area reaching the membrane protein of band 3. Hyperoxidized phospholipid bilayer increases non-specific cation permeability, and degradation of membrane proteins of several cation transporters induces Na^+^ influx and accumulation leading to the osmotic cell swelling in a Ca^2+^-free medium (Dwight and Hendry, 1996). Oxidative damage of band 3 protein regulating cell volume is supposed to accelerate erythrocyte swelling (López-Alarcóna et al., 2020). This speculation is supported by the superoxide-induced erythrocyte swelling in the hypoxanthine-xanthine oxidase system, that degrades several membrane proteins including band 3 (Uyesaka et al., 1992). The extent of erythrocyte swelling induced by tBHP application is reported to depend on external Ca^2+^ (Lisovskaya et al., 2008). This swelling in a Ca^2+^-free condition is greater than that observed in a Ca^2+^-containing medium, where the Gardos channel is activated by Ca^2+^-influx and results in cell dehydration and shrinkage counteracting cell swelling.

## Effects of Oxidative Stress on Erythrocyte Membrane

**FIGURE 4:**
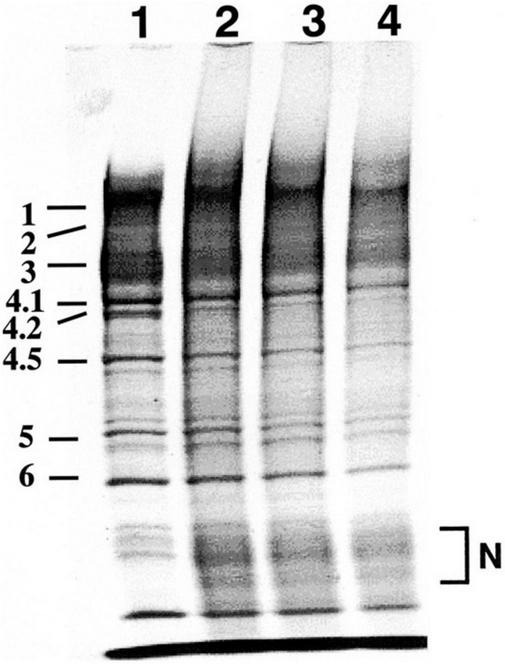
Effects of acute exposure to 1.0 mM tBHP on membrane protein was investigated by SDS polyacrylamide gel electrophoresis. 1, control untreated erythrocytes; 2, erythrocytes treated with tBHP (1.0 mM); 3, erythrocytes pretreated with 0.1 μM verapamil before exposure to tBHP (1.0 mM); 4, erythrocytes pretreated with 1.0 μM verapamil before exposure to tBHP (1.0 mM). Treatment with 1.0 mM tBHP caused degradation of membrane protein corresponding to spectrin (band 1, 2), band 3, band 4.2 and band 4.5 associated with the new appearance of the low-molecular-weight broadband as indicated by N. Degradation of band 3 known as membrane protein exchanging anions (Cl^–^ and HCO_3_^–^) and regulating cellular volume may contribute to tBHP-induced erythrocyte swelling. Although verapamil is a possible antioxidative agent, pretreatment with verapamil did not show any apparent protective effects [cited from Okamoto et al. (2004) with permission].

2,2′-azobis(2-amidinopropane) dihydrochloride-treated erythrocytes exhibit dark brown, which is compatible with the formation of methemoglobin due to oxidation of iron in heme from reduced ferrous (Fe^2+^) to oxidized ferric (Fe^3+^) state. Methemoglobin was assayed by standard spectrophotometric method (Okamoto et al., 2004), and an increase in methemoglobin of AAPH-treated erythrocytes indicates potent oxidation of heme iron and interaction of AAPH-generating ROS with oxyhemoglobin (Figure 3C). This phenomenon is confirmed in another oxidative stress model of human erythrocytes exposed to tBHP and superoxide anion (O_2_^•⁣–^) generated by hypoxanthine-xanthine oxidase reaction (Uyesaka et al., 1992; Okamoto et al., 2004). Standard high-performance liquid chromatography (HPLC) in our laboratory confirmed that exposure of intact erythrocytes to 50 mM AAPH reduced α- and γ-tocopherol to less than 50% and phosphatidylethanolamine (PE) to 80% of their initial level 120 min after starting incubation at 37°C (Mawatari et al., 2004). HPLC demonstrated a similar trend in the case of 1.0 mM tBHP application, and the reduction in PE was prevented by 0.1 mM ascorbate (Mawatari and Murakami, 2001). Therefore, the ROS-induced reduction in tocopherol and phospholipid mainly reflects a decrease in polyunsaturated fatty acids. Further, tBHP-induced membrane protein degradation was evident (Okamoto et al., 2004) especially in spectrin (band 1, 2), band 3, band 4.2, and band 4.5 in SDS polyacrylamide gel electrophoresis (Figure 4). A similar trend was confirmed in the case of membrane protein of erythrocytes exposed to extracellular superoxide anion (O_2_^•⁣–^) catalyzed by xanthine oxidase in the hypoxanthine-xanthine oxidase assay (Uyesaka et al., 1992).

Hemolysis is a final event of erythrocytes exposed to profound oxidative inflammation. Hemoglobin oxidation, a consumptive decline in antioxidant capacity, membrane phospholipid peroxidation and protein degradation evoke oxidative hemolysis in concert (López-Alarcóna et al., 2020). AAPH-induced time-dependent hemolysis was confirmed in our laboratory (Figure 3D). However, our two oxidative stress models using AAPH and tBHP did not induce the formation of the Heinz body that is aggregates of hemichromes (Okamoto et al., 2004). The oxidative inflammation caused by these agents alters dramatically the major determinants of erythrocyte deformability, i.e., erythrocyte swelling and oxidative crosslinking of methemoglobin and spectrin leading to the cytoskeletal conformational changes and loss of membrane flexibility (Murakami and Mawatari, 2003). The time courses of sigmoidal increase in MCV (Figure 3B), methemoglobin formation (Figure 3C), and hemolysis (Figure 3D) commonly show the maximum slope in the time segment of 60–120 min after starting incubation, whereas the time course of decline in the erythrocyte filterability has its maximum slope in 40–90 min after starting exposure. These indicate that erythrocyte filterability is a sensitive marker of cellular damage preceding hemolysis. Exogenous ROS generator application is drastic but adequate as an oxidative stress model using *ex vivo* erythrocytes in that these radical generators are chemically stable, and the rate of radical generation is dose-dependent and constant during the first several hours in the reaction mixture (Odashiro et al., 2014).

## Outcome of Erythrocytes Exposed to Oxidative Stress

Severe oxidative inflammation overwhelming antioxidative defense mechanisms induces irreversible erythrocyte damage leading to hemolysis as observed in the applications of AAPH and tBHP. However, persistent low-grade oxidative inflammation shortens the lifespan of circulating *in vivo* erythrocytes. Erythrocytes are sensitive to oxidative inflammation as a health indicator and inflamed erythrocytes undergo the programmed cell death known as eryptosis. Oxidative stress is one of the triggers of eryptosis, which is a suicidal erythrocyte death characterized by cell shrinkage, membrane blebbing, and phospholipid scrambling causing exposure of phosphatidylserine (PS) at the membrane surface. Oxidative stress activates non-selective cation channels, and massive Ca^2+^ entry causes the expression of Gardos channels and activates calpain and scramblase (Bernhardt et al., 2020). Calpain is a Ca^2+^-dependent protease degrading cytoskeleton, and scramblase is a Ca^2+^-binding enzyme breaking membrane phospholipid asymmetry and exerting membrane scrambling and PS exposure to the outer leaflet of the erythrocyte membrane bilayer. Eryptotic cell loses KCl and osmotically driven water leading to cell shrinkage. Ceramide, a product of sphingomyelinase from membrane sphingomyelin, also contributes to eryptosis. Externalized PS is a marker for splenic macrophages to engulf and degrade the eryptotic erythrocytes (Lang et al., 2014; Bissinger et al., 2019). Eryptosis aims to avoid hemolysis liberating damaged erythrocytes degradation product into circulation and reducing the bioavailability of nitric oxide (NO) profoundly. However, excessive eryptosis induces anemia that lowers systemic oxygen delivery. Therefore, proeryptotic and antieryptotic mechanisms are balanced to escape both hemolysis and anemia (Föller et al., 2008).

## Clinical Implications

Reactive oxygen species are byproducts of biological oxygen consumption and play a key role in oxidative stress. Our experimental data are the hemorheological and biochemical results of erythrocytes exposed to acute oxidative stress by excessive ROS derived from an exogenous source. It is inconclusive that these data are extrapolated to the persistent effects on circulating erythrocytes of ROS corresponding to 0.1–0.2% of utilized oxygen (Tahara et al., 2009). However, chronic, low-grade inflammation and increased oxidative stress coexist with lifestyle-related common diseases such as type 2 diabetes mellitus and hypertension, i.e., these are significant worldwide health burdens leading to systemic atherosclerosis and microangiopathy (Pouvreau et al., 2018). Impairment of erythrocyte deformability in animal models and patients with these common diseases is confirmed quantitatively in our laboratory (Ariyoshi et al., 2010; Saito et al., 2011; Odashiro et al., 2015; Arita et al., 2020). Oxidative inflammation is deeply involved also in the disease progression, a wide variety of complications, and therapeutic strategies of sickle cell disease (SCD), which is one of the most common inherited hemoglobin disorders characterized by sickle cell-mediated vaso-occlusion and intravascular hemolysis impairing bioavailability of NO. Further, eryptosis is accelerated in patients with diabetes, hypertension, and SCD, because oxidative stress is acting as proeryptotic event, and NO is an antieryptotic key regulator (Calderón-Salinas et al., 2011; Pinzón-Díaz et al., 2018; Nader et al., 2020).

### Diabetes Mellitus

Reactive oxygen species overproduction and chronic inflammation interact in the development and the progression of type 2 diabetes mellitus. Persistent hyperglycemia activates inflammatory cytokines, accelerates autoxidation of glucose, and promotes ROS generation and decrease in the antioxidative enzyme activity. Advanced glycation end products (AGE) derived from the Maillard reaction contribute to the oxidative inflammation (Newsholme et al., 2016; Luc et al., 2019). Acute exposure to AGE directly reduces erythrocyte deformability (Iwata et al., 2004), which is impaired also in Wistar-Kyoto rats treated with streptozotocin (STZ; 65 mg/kg) and in diabetic patients under treatment (Supplementary Figure 1). It is accepted widely that diabetic erythrocytes show reduced deformability and increased aggregability leading to prothrombotic tendency (Singh and Shin, 2009; Baskurt and Meiselman, 2013). MCV reflects erythrocyte size, and the mean corpuscular hemoglobin concentration (MCHC; g/dL) is a practical surrogate of internal viscosity. These two parameters in STZ-treated diabetic rats did not differ from respective parameters in control rats (Saito et al., 2011). The same was true in comparing MCV, MCHC, and erythrocyte density between diabetic and control patients (Arita et al., 2020). These indicate that internal viscosity, density, and cell geometry have no significant influence on the reduced deformability of diabetic erythrocytes. Accumulated evidence indicates biochemical and biophysical membrane abnormalities in diabetic erythrocytes, i.e., erythrocyte membrane fluidity is reduced in experimental and clinical diabetes (Waczulíkova et al., 2000; Pérez-Hernández et al., 2010), and altered compositions of fatty acid, phospholipid and cholesterol may underly the reduced diabetic erythrocyte membrane fluidity (Zeghari et al., 2000; Pérez-Hernández et al., 2010; Nunes and Pretorius, 2019).

### Hypertension

Hypertension is the main cause of cardiovascular diseases, and oxidative inflammation is deeply involved in the complex pathophysiology of hypertension along with sodium overloading, elevated sympathetic activity, an accelerated renin-angiotensin-aldosterone system that augments oxidative stress leading to endothelial dysfunction, vascular aging and remodeling in hypertensive patients (Reckelhoff and Romero, 2003; Guzik and Touyz, 2017). Spontaneously hypertensive rats (SHR) are recognized as an established model of human hypertension. Oxidative stress has been reported in SHR in these two decades, although it is not clear whether it is a primary involvement or a secondary result (de Champlain et al., 2004; Yanes et al., 2005). Accumulating evidence suggests that deformability of erythrocytes in SHR is impaired (Pezeshk and Dalhouse, 2000; Pérez-Hernández et al., 2010). This impairment is more significant in young prehypertensive SHR than in mature hypertensive SHR as confirmed in our laboratory (Supplementary Figure 2). The shear rate in resistance arterioles of SHR is elevated compared with that in control rats. This elevation is also greater in young SHR relative to mature SHR (Lominadze et al., 2001). Further, polycythemia is evident in SHR (Ariyoshi et al., 2010). These findings result in mechanical and oxidative stress affecting the erythrocyte membrane and reduced membrane fluidity in SHR (Pezeshk and Dalhouse, 2000; Pérez-Hernández et al., 2010). Shear-activated endothelium expresses inflammatory cytokines (interleukin-1β) and adhesion molecules (E-selectin), and inflammation evokes endothelial dysfunction (Huang and Eniola-Adefeso, 2012). Therefore, oxidatively and mechanically impaired erythrocyte deformability and elevated vascular resistance assist the development of hypertension in SHR. Impaired erythrocyte deformability is confirmed in clinical hypertension, and this impairment is proportional to the mean blood pressure in hypertensive patients under treatment. Among the three major determinants of deformability, MCHC representing internal viscosity and MCV reflecting cell size showed no significant differences between hypertensive patients and normotensive controls (Odashiro et al., 2015). These imply that erythrocyte membrane property is involved in the impaired deformability contributing in part to the pathophysiology of clinical hypertension (Tsuda, 2010).

### Sickle Cell Disease

Sickle cell disease is an autosomal recessive disease affecting millions of people worldwide. This hereditary hemoglobinopathy is caused by a single mutation of the β-globin gene producing abnormal hemoglobin called sickle hemoglobin (HbS). Erythrocyte sickling occurs in the deoxygenated condition by polymerization of HbS (Supplementary Figure 3). This HbS polymerization and dehydration elevating internal viscosity are the primary pathophysiologies of SCD characterized by vaso-occlusive crisis, endothelial dysfunction, vascular injury, hemolytic anemia, and multiple organ damage. Sickle erythrocytes are heterogeneous in terms of cellular morphology, density, viscosity, fragility, and deformability. The mechanisms of such phenotype complexity remain unclear. The current paradigm is that SCD is not merely a rheological disease and that oxidative inflammation is deeply involved in its pathophysiology. Production of ROS is augmented and counteracting antioxidative capacity is consumed by repetitive microcirculatory occlusion-reperfusion insults. Such redox imbalance is reported in patients with SCD (Biswal et al., 2019; Caprari et al., 2019). Apart from curative treatment with hematopoietic stem cell transplantation and gene therapy, hydroxyurea (HU) is a first-line therapy in symptomatic patients with SCD (Carden and Little, 2019). Administration of HU produces fetal hemoglobin (HbF) leading to an inhibition of HbS polymerization and an improvement of erythrocyte deformability. However, an increase in HbF by HU alone is variable, and long-term use of HU may induce myelosuppression and chromosome changes. The combination therapy of HU and recombinant erythropoietin potentiates the elevation of HbF and rheological improvement, which was confirmed for the first time by the nickel mesh filtration technique (Rodgers et al., 1993). Treatment with HU also restores the redox imbalance, indicating that oxidative inflammation plays a significant role in the pathogenesis of SCD (Renó et al., 2020). Nowadays, coronavirus disease 2019 (COVID-19) is spreading worldwide, and this pandemic viral infection alters the rheological properties of blood cells including erythrocytes, that persists for months after hospitalization (Kubánková et al., 2021). Patients with SCD are at high risk of COVID-19. Because sickling easily occurs in COVID-19-induced hypoxic conditions.

## Conclusion

**FIGURE 5:**
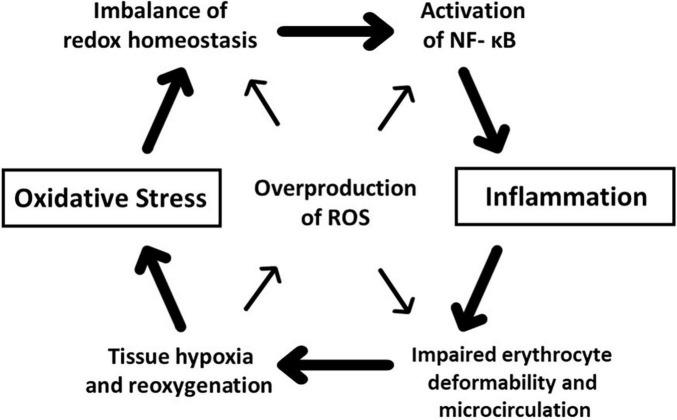
Schematic illustration of the hypothetical positive feedback explaining the concept of oxidative inflammation. Reactive oxygen species (ROS) overproduced by oxidative stress play a central role in this vicious cycle. ROS-injured cellular components trigger systemic inflammation *via* activation of transcriptional factors such as nuclear factor κ-B (NFκ-B). Inflammatory endothelial dysfunction impairs erythrocyte deformability. Further, ROS-impaired erythrocyte deformability disturbs microcirculation and causes tissue hypoxia, and reoxygenation promotes overproduction of ROS and imbalance of redox homeostasis. Redox-sensitive NF-κB activation sustains systemic inflammation, and inflamed immune cells generate ROS maintaining the putative positive feedback.

The physiological level of ROS is not only the inevitable byproducts of cellular metabolism but also the signaling molecules enhancing antioxidative defense mechanisms and initiating an inflammatory response to restore the ROS-injured cellular components. As a cellular model of acute oxidative inflammation, exposure of *ex vivo* human erythrocytes to ROS generators impairs the deformability seriously by chain-reacting auto-oxidation causing methemoglobin formation, membrane phospholipid peroxidation, protein degradation, and subsequent hemolysis. This impairment of circulating erythrocyte deformability *in vivo* disturbs systemic microcirculation and causes profound tissue hypoxia. Although there is still a gap between the chronic overproduction of ROS under the redox imbalance observed in patients with diabetes, hypertension, SCD, and acute oxidative stress caused by overwhelming ROS generation, the positive feedback amplifying oxidative inflammation is supposed to trigger the eryptosis to avoid intravascular hemolysis and sustain the pathophysiology of aforementioned diseases (Figure 5), i.e., rigid erythrocytes induce tissue hypoxia, and reoxygenation results in endogenous ROS overproduction and accelerates systemic inflammation *via* activation of the redox-sensitive nuclear transcriptional factor of NF-κB. Therefore, the concept of reversing the oxidative-inflammatory positive feedback may offer the new therapeutic targets to improve erythrocyte rheology in many clinical conditions.

## Author Contributions

TM had an initial concept of this review article and made manuscript writing. MH was chief of the rheological laboratory, engaged in data acquisition and maintenance of erythrocyte filtration apparatus, and made critique and advice on the manuscript revision. SM was chief of the biochemical laboratory and is involved in the maintenance of the high-performance liquid chromatography separating all the major phospholipids in the erythrocyte membrane. TF was the project leader, applied to the funding, and supervised the team collaboration. All authors approved the manuscript submission to this journal.

## Conflict of Interest

SM and TF was employed by Institute of Rheological Function of Foods Co., Ltd. The remaining authors declare that the research was conducted in the absence of any commercial or financial relationships that could be construed as a potential conflict of interest.

## Publisher’s Note

All claims expressed in this article are solely those of the authors and do not necessarily represent those of their affiliated organizations, or those of the publisher, the editors and the reviewers. Any product that may be evaluated in this article, or claim that may be made by its manufacturer, is not guaranteed or endorsed by the publisher.

## Supplementary Material

Supplementary Figure 1 Representative *P(t)-Q(t)* relationships during continuous filtration experiments using HEPES-buffered control saline and erythrocyte suspensions. **(A)** *P(t)-Q(t)* relationships of gravity filtration using saline (○, ◇) and suspension of erythrocytes obtained from control Wistar-Kyoto (WKY) rats (△, □) and those from streptozotocin-treated diabetic WKY rats (▲, ■). The hematocrit of erythrocyte suspension was 2.0%, and the pore size of the nickel mesh filter was uniformly 3.85 μm for rat erythrocytes. **(B)** *P(t)-Q(t)* relationships of control saline (lines without symbols) and erythrocyte suspensions of non-diabetic subjects (○, △, □). **(C)** *P(t)-Q(t)* relationships of control saline (lines without symbols) and suspensions of erythrocytes obtained from diabetic patients associated with obesity (, ▲, ■, ◆) or not (○, △, □, ◇). The hematocrit of erythrocyte suspension was 3.0%, and the pore size of the nickel mesh filter was uniformly 4.94 μm for human erythrocytes. Erythrocyte filterability is impaired in diabetic rats and diabetic obese patients [cited from Saito et al. (2011) with permission].

Supplementary Figure 2 Representative *P(t)-Q(t)* relationships of control saline (◯, ) and suspensions of erythrocytes obtained from spontaneously hypertensive rats (SHR) and age-matched control Wistar-Kyoto rats (WKY). Erythrocyte filterabilities of SHR aged 7 (), 13 (), and 18 () weeks are compared with those in WKY aged 7 (), 13 (), and 18 () weeks, respectively. The hematocrit of erythrocyte suspension was 2.0%, and the pore size of the nickel mesh filter was uniformly 3.88 μm. Erythrocyte filterability in SHR is lower than that in the age-matched WKY at any given age. Prehypertensive SHR aged 7 weeks () show greater impairment of erythrocyte filterability than mature hypertensive SHR aged 18 weeks (). SHR were purchased from Charles River Japan [cited from Ariyoshi et al. (2010) with permission].

Supplementary Figure 3 A scanning electron microscopic observation of erythrocytes obtained from a patient with sickle cell disease (SCD) that is a hemoglobinopathy producing sickle hemoglobin (homozygous HbS) based on a single mutation in the sixth codon of the β-globin gene (βGlu6Val). Sickle erythrocyte shows normal biconcave disk shape in the oxygenated state **(A)**, but sickling occurs in the deoxygenated state **(B)**. Scale bars indicate 3 μm [reproduced by courtesy of Dr. Nobuhiro Uyesaka and cited from Maruyama et al. (2020) with permission].
