# Supplementary figures and images for: Rheological Abnormalities in Human Erythrocytes Subjected to Oxidative Inflammation
Source: Front Physiol. 2022 Feb 23;13:837926. doi: 10.3389/fphys.2022.837926 (PMC8905344; doi:10.3389/fphys.2022.837926)

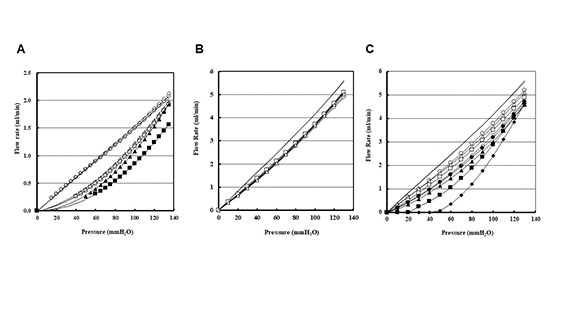

Supplement: Supplementary Figure 1 — Representative P(t)-Q(t) relationships during continuous filtration experiments using HEPES-buffered control saline and erythrocyte suspensions. (A) P(t)-Q(t) relationships of gravity filtration using saline (○, ◇) and suspension of erythrocytes obtained from control Wistar-Kyoto (WKY) rats (△, □) and those from streptozotocin-treated diabetic WKY rats (▲, ■). The hematocrit of erythrocyte suspension was 2.0%, and the pore size of the nickel mesh filter was uniformly 3.85 μm for rat erythrocytes. (B) P(t)-Q(t) relationships of control saline (lines without symbols) and erythrocyte suspensions of non-diabetic subjects (○, △, □). (C) P(t)-Q(t) relationships of control saline (lines without symbols) and suspensions of erythrocytes obtained from diabetic patients associated with obesity (, ▲, ■, ◆) or not (○, △, □, ◇). The hematocrit of erythrocyte suspension was 3.0%, and the pore size of the nickel mesh filter was uniformly 4.94 μm for human erythrocytes. Erythrocyte filterability is impaired in diabetic rats and diabetic obese patients [cited from Saito et al. (2011) with permission]. [file Image_1.JPEG]

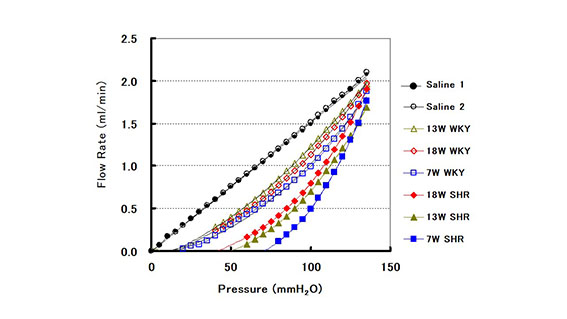

Supplement: Supplementary Figure 2 — Representative P(t)-Q(t) relationships of control saline (◯, ) and suspensions of erythrocytes obtained from spontaneously hypertensive rats (SHR) and age-matched control Wistar-Kyoto rats (WKY). Erythrocyte filterabilities of SHR aged 7 (), 13 (), and 18 () weeks are compared with those in WKY aged 7 (), 13 (), and 18 () weeks, respectively. The hematocrit of erythrocyte suspension was 2.0%, and the pore size of the nickel mesh filter was uniformly 3.88 μm. Erythrocyte filterability in SHR is lower than that in the age-matched WKY at any given age. Prehypertensive SHR aged 7 weeks () show greater impairment of erythrocyte filterability than mature hypertensive SHR aged 18 weeks (). SHR were purchased from Charles River Japan [cited from Ariyoshi et al. (2010) with permission]. [file Image_2.JPEG]

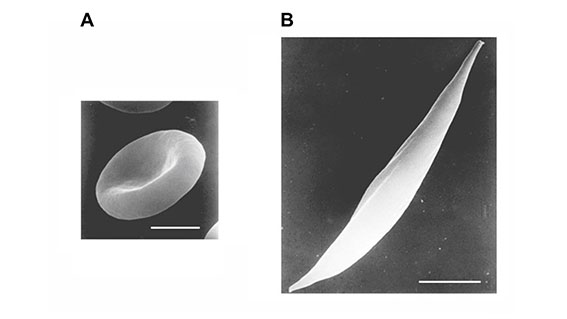

Supplement: Supplementary Figure 3 — A scanning electron microscopic observation of erythrocytes obtained from a patient with sickle cell disease (SCD) that is a hemoglobinopathy producing sickle hemoglobin (homozygous HbS) based on a single mutation in the sixth codon of the β-globin gene (βGlu6Val). Sickle erythrocyte shows normal biconcave disk shape in the oxygenated state (A), but sickling occurs in the deoxygenated state (B). Scale bars indicate 3 μm [reproduced by courtesy of Dr. Nobuhiro Uyesaka and cited from Maruyama et al. (2020) with permission]. [file Image_3.JPEG]
